# LncRNA-MSTRG.19083.1 Targets NTRK2 as a miR-429-y Sponge to Regulate Circadian Rhythm via the cAMP Pathway in Yak Testis and Cryptorchidism

**DOI:** 10.3390/ijms252413553

**Published:** 2024-12-18

**Authors:** Tianan Li, Qiu Yan, Jinghong Nan, Xue Huang, Ruiqing Wang, Yong Zhang, Xingxu Zhao, Qi Wang

**Affiliations:** 1 College of Veterinary Medicine, Gansu Agriculture University, Lanzhou 730070, China; 17899315032@163.com (T.L.); 18776410232@163.com (Q.Y.); n18894000502@163.com (J.N.); 13028737980@163.com (X.H.); 17352255912@163.com (R.W.); zhangyong@gsau.edu.cn (Y.Z.); 2 Gansu Key Laboratory of Animal Generational Physiology and Reproductive Regulation, Lanzhou 730070, China

**Keywords:** yak, cryptorchidism, LncRNA, NTRK2, circadian rhythm

## Abstract

Long noncoding RNAs (LncRNAs) play essential roles in numerous biological processes in mammals, such as reproductive physiology and endocrinology. Cryptorchidism is a common male reproductive disease. Circadian rhythms are actively expressed in the reproductive system. In this study, a total of 191 LncRNAs were obtained from yak testes and cryptorchids. Then, we identified NTRK2’s relationship to circadian rhythm and behavioral processes. Meanwhile, the ceRNA (LncRNA-MSTRG.19083.1/miR-429-y/NTRK2) network was constructed, and its influence on circadian rhythm was revealed. The results showed that NTRK2 and LncRNA-MSTRG.19083.1 were significantly upregulated, and miR-429-y was obviously decreased in cryptorchid tissue; NTRK2 protein was mainly distributed in the Leydig cells of the testis. In addition, the upregulation of the expression level of miR-429-y resulted in the significant downregulation of LncRNA and NTRK2 levels, while the mRNA and protein levels of CREB, CLOCK, and BMAL1 were significantly upregulated; the knockdown of miR-429-y resulted in the opposite changes. Our findings suggested that LncRNA-MSTRG.19083.1 competitively binds to miR-429-y to target NTRK2 to regulate circadian rhythm through the cAMP pathway. Taken together, the results of our study provide a comprehensive understanding of how the LncRNA-miRNA-mRNA networks operate when yak cryptorchidism occurs. Knowledge of circadian-rhythm-associated mRNAs and LncRNAs could be useful for better understanding the relationship between circadian rhythm and reproduction.

## 1. Introduction

Cryptorchidism is one of the main causes of infertility in male yaks [1] and clinically manifests in one or both non-palpable testes in the scrotum. Due to the failure of testicular descent into the scrotum, the temperature of the testes retained in the inguinal canal or abdominal cavity increases, the blood oxygen content decreases, the microenvironment of spermatogenesis changes, and the normal spermatogenesis is interrupted, eventually leading to infertility [2,3,4].

In recent years, with the rapid development of modern molecular biology technology and the second generation of high-throughput parallel sequencing technology, researchers have found that noncoding RNA (ncRNA), as a gene expression regulator, participates in the regulation of gene expression at transcriptional, post-transcriptional, epigenetic, and other levels [5]. Long noncoding RNAs (LncRNAs) represent transcripts that have no coding potential, generally over 200 nt in length [6]. LncRNAs, as critical regulators in normal and disease development, provide new clues for delineating the molecular regulation in male germ cell development [7]. The testis is an organ with strong transcriptional activity, and its development is regulated by many ncRNAs. In addition, seminiferous epithelium, Sertoli cells (SCs), and Leydig cells (LCs) have hypercyclic (multi-hour), mesocyclic (multi-day), and seasonal periodicity [8]. Previous studies have shown that the natural conception rate in mammals is rhythmic, which is related to seasonal variations, light cycles, monthly average sunshine hours, temperature, and humidity [9]. LncRNA has long been regarded as transcriptional noise due to its inability to encode proteins, poor conservatism, and low expression level, as well as the limited evidence of its function [10]. In recent years, an increasing amount of evidence has demonstrated that many LncRNAs are involved in the regulation of the mammalian testis, and this regulation is dynamic [11]. Neonatal and adult mouse testes have demonstrated dynamic LncRNA expression and exhibited associations with epigenetic modifications and evolutionarily conserved elements. Furthermore, the expression profile of LncRNAs has been reported to be tissue- or cell-specific [12]. In addition, it has been confirmed that semen quality varies with time and season in both humans and rodents, with the highest sperm count, concentration, and normal morphology in semen samples collected in the early morning [13,14]. Similarly, other studies reported better sperm parameters in spring or winter, while another study [15] has provided evidence for a higher prevalence of samples with normal sperm pH during spring and a higher volume of sperm in winter. The circadian rhythm, or circadian clock, mainly refers to the intrinsic mechanisms that respond to the ambient light/dark cycle, which has an oscillating period of approximately 24 h, responds to abiotic or biotic factors, and coordinates biological processes that adapt to daily environmental changes [16,17]. The intrinsic circadian clock is self-sustaining, through the control of negative feedback loops of the molecular clock [18], and exerts its influence through the control of metabolism, endocrine and immune function, and behavior [19]. The core clock genes include brain and muscle aryl hydrocarbon receptor nuclear transporter-like protein 1 (BMAL1), circadian locomotor output cycles kaput (CLOCK), and period (Per) [20]. Mutations in some key regulators of the circadian clock are associated with reduced male fertility in animal models. Mice with homozygous mutations in the *Clock* gene have reduced male fertility. Furthermore, it has been suggested that *Clock* can interact with *RANBP9* and bind several key transcripts in mouse spermatogenesis [21]. Mutations in another central circadian *Clock* gene, Bmal1, are associated with male infertility. Male knockout Bmal1 mice had low testosterone and high luteinizing hormone concentrations, suggesting steroidogenesis impairment in the testes and other steroidogenic tissues [22].

NTRK2, namely TrkB too, is a receptor tyrosine kinase that is the receptor for brain-derived neurotrophic factor (BDNF) [23]. By binding to its ligand, the NTRK2 receptor activates downstream signaling pathways that promote nerve growth, synaptic plasticity, and neuronal survival. The role of NTRK2 receptors is particularly prominent in the suprachiasmatic nucleus (SCN) [24]. It has been shown that the loss or dysfunction of TrkB leads to a disturbance of the circadian cycle, which manifests itself as a lengthening or shortening of the period of the circadian clock or even a loss of the regularity of the circadian rhythm [25]. The occurrence of cryptorchidism is usually related to the abnormality of testicular descent during the embryonic period, mainly related to the disorder of the hypothalamic–pituitary–testicular axis, hormone secretion imbalance, and the abnormal action of nerve growth factor [26,27]. NTRK2 may be involved in the development of nerve tissue or blood vessels in the testis, especially during testicular decline, and activation may promote the secretion of local nerve growth factor, thereby regulating the development of testicular tissue [28]. NTRK2 may affect the development of the testis by regulating the specific signaling pathways of related hormones, and cryptorchidism often has abnormalities in certain hormone signaling pathways [29,30].

In this present manuscript, our RNA-seq results have shown that the transcription level of NTRK2 in cryptorchidism is significantly increased. It is worth noting that previous studies have found that NTRK2 was involved in multiple biological processes such as reproduction, sexual behavior, and circadian rhythm [25,31,32]. Although the role and expression mechanisms of NTRK2 in cryptorchidism have not been fully studied, NTRK2 may be involved in the development of cryptorchidism through multiple mechanisms such as neural growth factor signaling pathways, hormonal regulation, and regulation of the function of neurons in the testes or circadian rhythms. Further studies will help clarify the specific role NTRK2 plays in this biological process and whether it may be a new target for the treatment of cryptorchidism or related diseases. Based on the above research, this study attempted to clarify the correlation between NTRK2 and the occurrence of cryptorchidism and male reproduction, and the targeting relationship between NTRK2 and LncRNA was explored, and the molecular mechanism of LncRNA-miRNA-NTRK2 regulation of circadian rhythm was revealed.

## 2. Results

### 2.1. Identifying Differentially Expressed LncRNAs of LncRNA Sequencing

**Figure 1:**
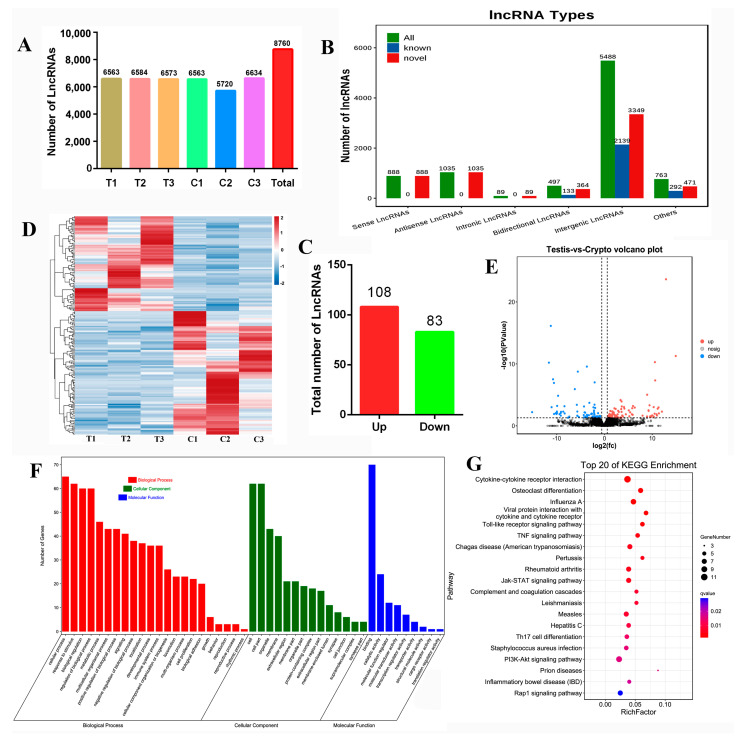
Analysis of differentially expressed LncRNA: (**A**) LncRNA distribution in the testis group and the cryptorchidism group; (T, testis; C, cryptorchidism); (**B**) number of LncRNA types; (**C**) total number of LncRNAs that were differentially expressed in the testis and the cryptorchidism group; the red color represents the number of LncRNAs upregulated in cryptorchidism compared to normal testes, the green color represents the number of LncRNAs decreased. (**D**) cluster heat map analysis of testis and cryptorchidism; (**E**) LncRNA differentially expressed volcano map; (**F**) GO analysis of 191 differentially expressed LncRNAs; (**G**) KEGG enrichment analysis.

Averages of 256,433,836 and 282,719,358 total reads were obtained for the normal testis (*n* = 3) and cryptorchidism (*n* = 3) libraries, respectively. After filtering, we finally obtained 255,690,178 (99.71%) and 281,861,776 (99.69%) LncRNA clean tags, respectively. A total of 8760 LncRNAs were identified (Figure 1A). According to the position of new LncRNAs relative to protein-coding genes on the genome, novel LncRNAs can be divided into six categories (Figure 1B). Of 888 sense-overlapping LncRNAs, all were unknown (100%); of 1035 antisense LncRNAs, all were novel LncRNAs (100%); of 89 intronic LncRNAs, all were novel LncRNAs (100%); of 497 bidirectional LncRNAs, 133 were known (26.76%) and 364 were novel LncRNAs (73.24%); of 5488 intergenic LncRNAs, 2139 were known (38.98%) and 3349 were novel LncRNAs (61.02%). There are 763 other LncRNAs. After applying stringent filtering criteria (*p* < 0.05 and log2(fold change) > 1) by pairwise intergroup alignment, a total of 191 differentially expressed LncRNAs were identified, of which 108 LncRNAs (56.54%) were upregulated and 83 LncRNAs were downregulated (43.46%) (Figure 1C). Hierarchical clustering was performed on the expression patterns of differentially expressed LncRNAs, and the clustering results were presented using heat maps. The clustering results of differentially expressed LncRNAs are shown in Figure 1D. According to the significant differences in LncRNA among the comparison groups, we performed volcano map analysis to show the different LncRNAs between the comparison groups. In the graph, the closer the LncRNA is to the two ends, the greater the degree of difference (Figure 1E). GO analysis found 191 differentially expressed LncRNAs (Figure 1F). Biological process GO items are mainly related to transcriptional regulation, response stimulus, metabolic process, and developmental process. Cell component GO focuses on cell parts, organelles, and cell membranes. The GO articles of molecular function are mainly concentrated in protein binding, catalytic activity, and regulation of molecular function. The top 20 signaling pathways with significant enrichment of differentially expressed LncRNAs are shown in Figure 1G. Among them, the significant differences are cytokine–cytokine receptor interaction, osteoclast differentiation, the toll-like receptor signaling pathway, and the TNF signaling pathway.

### 2.2. GO and KEGG Analysis of Differentially Expressed Genes (Circadian Rhythms)

**Figure 2:**
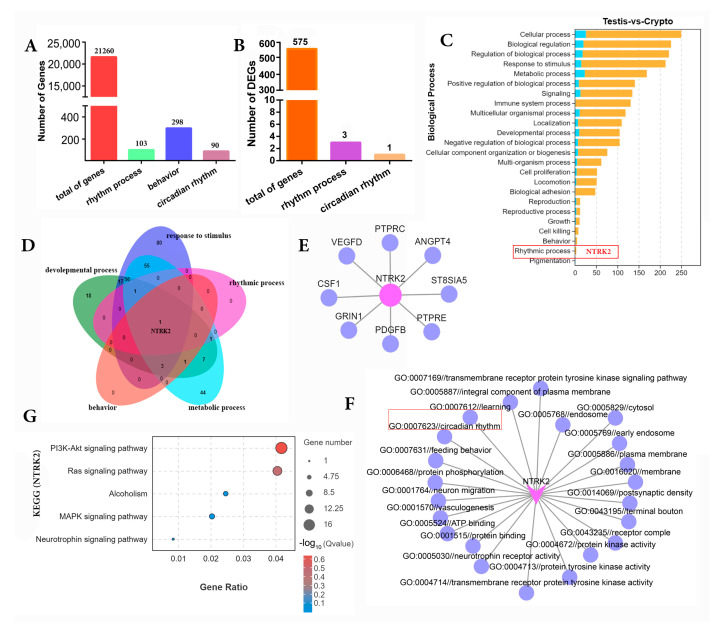
GO and KEGG analysis of differentially expressed genes (circadian rhythms): (**A**) genes involved in circadian activity were analyzed in testis and cryptorchids; (**B**) analysis of differentially expressed genes involved in circadian rhythms in testicular and cryptorchidism; (**C**) biological process analysis of circadian rhythm-related differential genes; (**D**) screening for circadian rhythm-related differential genes using Veen map; (**E**) interaction gene analysis of NTRK2; (**F**) functional analysis of NTRK2 involved in the GO enrichment process; (**G**) NTRK2 is involved in the enrichment signaling pathway.

A total of 21,620 mRNAs were identified from the mRNA-seq analysis of testis and cryptorchids, of which 103 were involved in rhythmic processes, 298 were involved in behavioral processes, and 90 were involved in circadian rhythm processes (Figure 2A). Based on log2(fold change) > 1 and FDR < 0.05, a total of 575 differentially expressed genes were identified, of which 3 were involved in the biological rhythm process and 1 was involved in circadian rhythms (Figure 2B). GO enrichment analysis showed that most DEGs were mainly involved in the biological processes, molecular function, and cellular component; in response to stimulus and developmental process, only one of these genes is involved in the circadian rhythms (Figure 2C). A Venn diagram for further screening and analysis showed that *NTRK2* is involved in the circadian rhythms (Figure 2D), and PPI analysis showed that NTRK2 mainly interacts with eight proteins (Figure 2E). Further analysis of GO function shows that NTRK2 is mainly involved in 22 specific functions (Figure 2F). Pathway analysis shows that NTRK2 is mainly involved in the PI3K-Akt signaling pathway, Ras signaling pathway, alcoholism, MAPK signaling pathway, and neurotrophin signaling pathway (Figure 2G).

### 2.3. Validation of Differentially Expressed LncRNAs and miRNA

**Figure 3:**
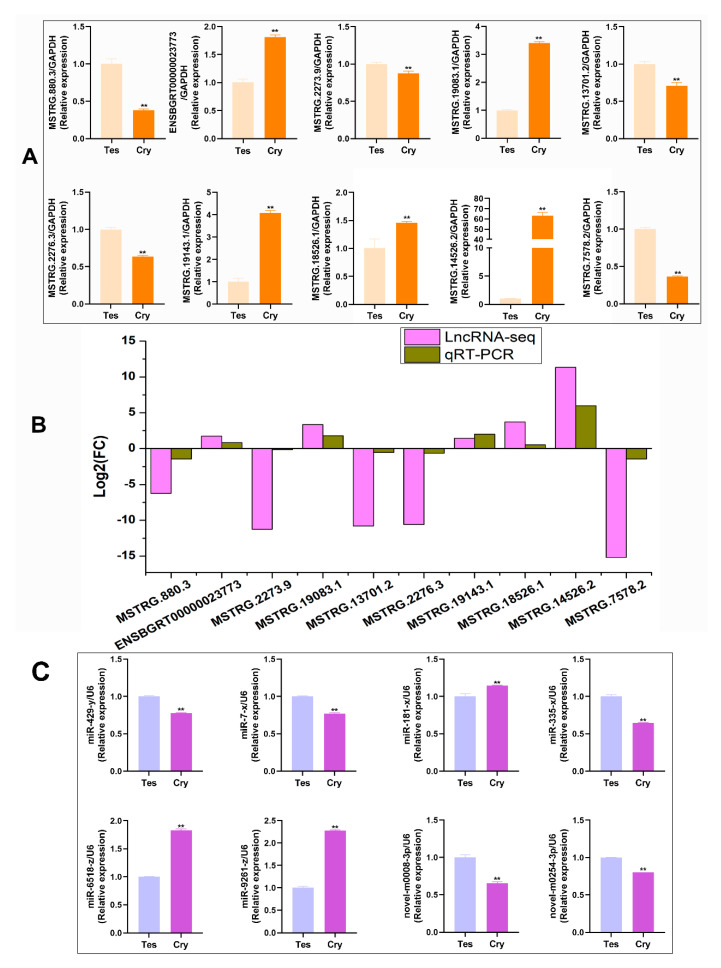
Verification of differentially expressed LncRNAs in the testis and cryptorchids: (**A**) differentially expressed LncRNAs were detected with qRT-PCR, *n* = 3, mean ± SD, ** p < 0.01; (**B**) LncRNA sequencing analysis; (**C**) differentially expressed miRNAs were detected with qRT-PCR, *n* = 3, mean ± SD, ** *p* < 0.01.

Real-time PCR was performed to validate the results of the 10 differentially expressed LncRNAs and 8 miRNAs. The PCR results suggested that all LncRNAs were differentially expressed in the testis and cryptorchids. Compared with the testis, five LncRNAs were significantly upregulated and five LncRNAs were significantly downregulated in the cryptorchidism (Figure 3A). The expression trends of 10 LncRNAs followed those of LncRNA-seq in terms of significant differences (*p* < 0.05 or *p* < 0.01), indicating that the LncRNA-seq results were reliable for identifying LncRNAs (Figure 3B). The PCR results suggested that all miRNAs were differentially expressed in the testis and cryptorchids. Compared with the testis, three miRNAs were significantly upregulated and five miRNAs were significantly downregulated in the cryptorchidism (Figure 3C).

### 2.4. Validation of Differentially Expressed mRNAs

**Figure 4:**
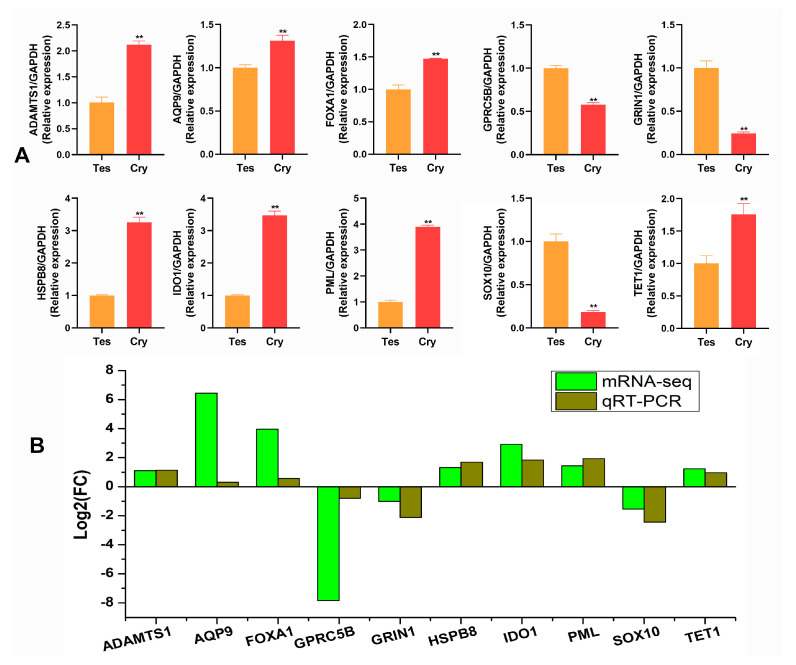
Differential expression of genes was verified in the testis and cryptorchids of the yaks: (**A**) differentially expressed mRNAs were detected with qRT-PCR, *n* = 3, mean ± SD, ** p < 0.01; (**B**) mRNA sequencing analysis.

Ten differentially expressed mRNAs were randomly selected to verify the accuracy of the mRNA-seq results with qRT-PCR analysis. The results suggested that all mRNAs were differentially expressed in the testis and cryptorchids: seven mRNAs were significantly upregulated and three mRNAs were significantly downregulated in the cryptorchidism compared with normal testis (Figure 4A). The expression trends of the 10 mRNAs followed those of mRNA-seq in terms of significant differences (*p* < 0.05 or *p* < 0.01), indicating that the mRNA-seq results were reliable for identifying mRNAs (Figure 4B).

### 2.5. Validation of the mRNA and Protein Levels of NTRK2 in Testis and Cryptorchidism

**Figure 5:**
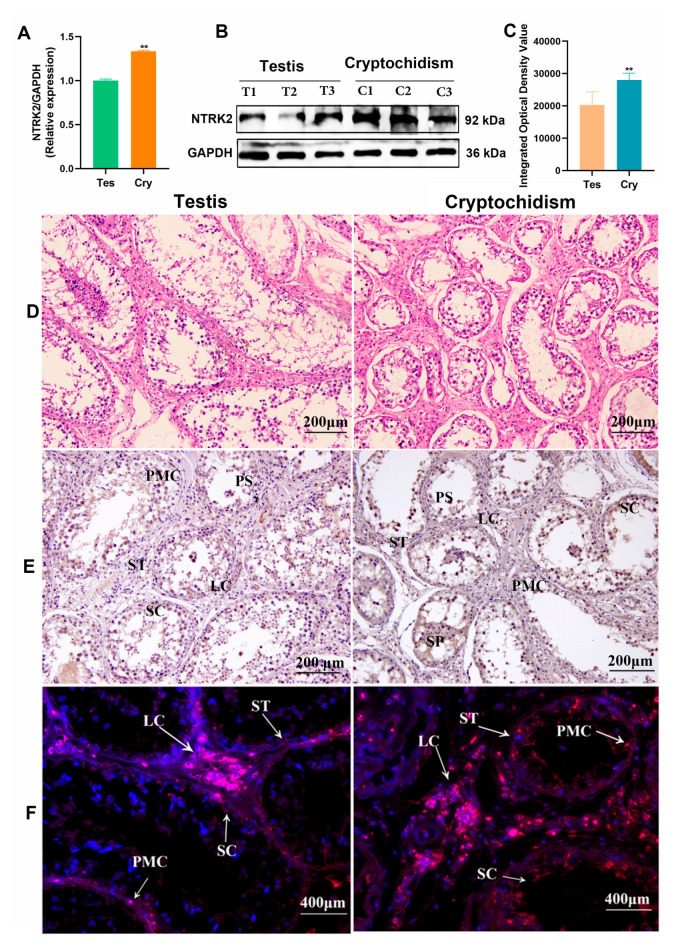
NTRK2 expression pattern analysis in testis and cryptorchids: (**A**–**C**) mRNA and protein expression levels of NTRK2 were analyzed using qPCR and Western blot, *n* = 3, mean ± SD, ** p < 0.01; (**D**) H&E staining was used to analyze the morphology and structure of testis and cryptorchids; (**E**,**F**) protein distribution of NTRK2 was stained by immunohistochemistry and immunofluorescence in testis and cryptorchids. LC: Leydig cells, SC: Sertoli cells, SP: spermatogonium, PS: primary spermatocyte, ST: seminiferous tubule, PMC: peritubular myoid cells.

To further verify the function of NTRK2, qRT-PCR, Western blot, immunohistochemistry, and immunofluorescence were used to verify the expression patterns of NTRK2 in testis and cryptorchids. The PCR results showed that compared with the testis, the mRNA level of *NTRK2* in cryptorchids was significantly upregulated (*p* < 0.01) (Figure 5A), and the NTRK2 protein was increased significantly in cryptorchidism (*p* < 0.01, Figure 5B,C). The H&E results revealed that the tubules are intact and tightly arranged in normal testis; conversely, the cryptorchidism group manifested an array of histologic aberrations, including disrupted seminiferous tubules with severe atrophy alongside diffuse necrosis degeneration of germinal cells and loose and diffuse connective tissue (Figure 5D). The IHC results showed that there were stronger positive signals for the NTRK2 protein in vascular endothelium, Leydig cells, Sertoli cells, and primary spermatocytes; medium positive signals in spermatogonia; and weakly positive signals in peritubular myoid cells from testis tissues. However, in cryptorchid tissues, there were stronger positive signals for the NTRK2 protein in Sertoli cells, Leydig cells, and primary spermatocytes; medium positive signals in spermatogonia; and weak positive signals in peritubular myoid cells (Figure 5E). The IF results showed that the NTRK2 protein’s stronger signals are focused on Leydig cells and Sertoli cells in the testis. However, in cryptorchid tissues, the stronger NTRK2 protein signals are mainly distributed in Leydig cells, with weak positive expression being found in Leydig cells and peritubular myoid cells (Figure 5F and Figure S1).

### 2.6. Relationships Between LncRNA/NTRK2 and miRNA

**Figure 6:**
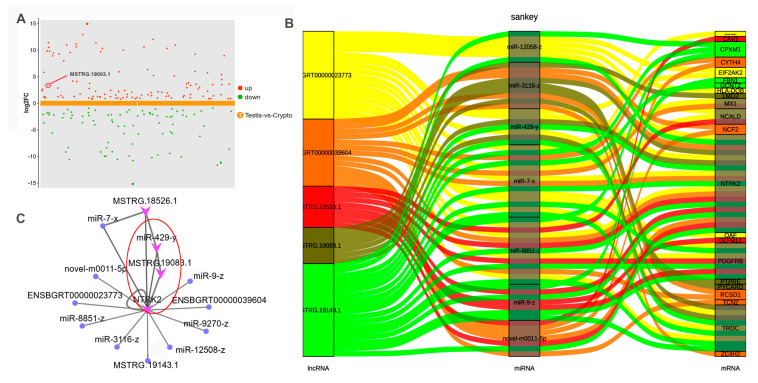
Targeting relationship between *NTRK2* and LncRNAs/miRNAs: (**A**) the scatter plot revealed the expression level of LncRNAs; (**B**,**C**) the network map and mulberry map reveal the targeting relationship between NTRK2 and LncRNAs and miRNAs. –– means Gene unknown.

To further explore whether *NTRK2* is regulated by LncRNA, the targeting relationship between *NTRK2* and LncRNA/miRNA was analyzed through scatter plots and mulberry plots. The results showed that five LncRNAs (MSTRG.18526.1, ENSBGRT00000039604, MSTRG.19143.1, ENSBGRT00000023773, and MSTRG.19083.1) and seven miRNAs (novel-m0011-5p, miR-12058-z, miR-429-y, miR-3116-z, miR-7-x, miR-8851-z, and miR-9-z) were associated with 22 mRNAs, among which *NTRK2* has a targeting relationship with all miRNAs and LncRNAs (Figure 6A–C).

### 2.7. Verification of Leydig Cells and Validation of Predicted Targeting of NTRK2 Gene, LncRNA-MSTRG.19083.1, and miR-429-y

**Figure 7:**
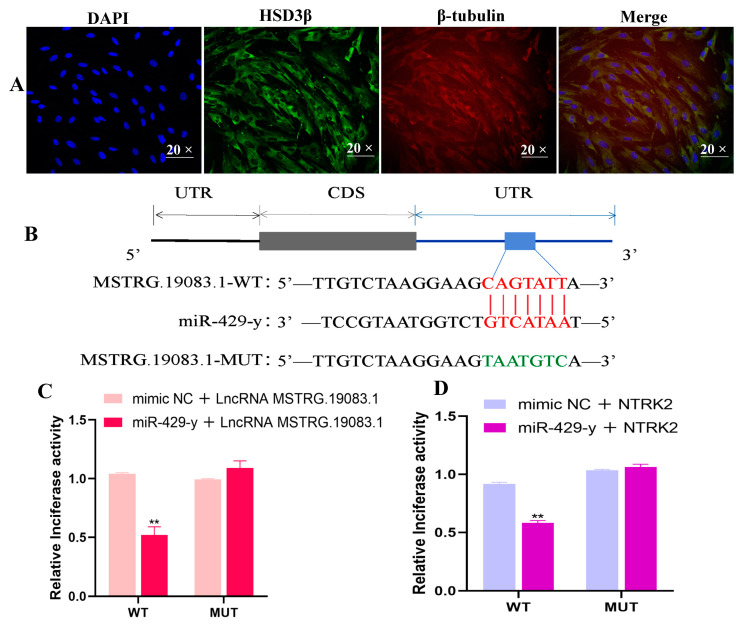
Between target gene NTRK2 and LncRNA and miR-429-y, (**A**) IF staining identified isolated yak LCs using antibodies against HSD3β (green) and β-tubulin (red), with magnification, 20×; (**B**,**C**) binding site of LncRNA-MSTRG.19083.1, NTRK2, and miRNA-429-y; (**D**) Luciferase activity in 293T cells after co-transfection with mimics of miRNA-429-y (100 nM) or mimic NC (100 nM) and pmirGLO-LncRNA-MSTRG.19083.1/NTRK2 3′-UTR-WT (400 ng) or pmirGLO-LncRNA-MSTRG.19 083.1/NTRK2 3′-UTR-MUT (400 ng). Values represent mean ± SD; *n* = 3, ** p < 0.01.

We used one key protein (HSD3β) to identify the purity of primary yaks’ LCs, and β-tubulin was used to stain the cytoskeleton. According to the IF results, the nuclei are stained blue and the β-tubulin is stained red; the HSD3β is stained green separately. Almost all cells in the field of view could be labeled with green fluorescence. HSD3β is a cytoplasmic protein that shows the high purity of the separation and purification of primary LCs, which can be used in subsequent cell transfection (Figure 7A). Binding seed sequences were in alignment with the NTRK2 3′-UTR and miR-429-y group and the LncRNA-MSTRG.19083.1 3′-UTR and miR-429-y group and were predicted using TargetScan (Version 7.2) and miRanda (V0.10.73) bioinformatics software. The LncRNA-MSTRG.19083.1-MUT group showed seven mutated nucleotides in the LncRNA-MSTRG.19083.1 3′-UTR (Figure 7B). The activity of the luciferase reporter in the LncRNA-MSTRG.19083.1 3′-UTR-WT + miR-429-y mimic group was significantly lower than that in the LncRNA-MSTRG.19083.1 3′- UTR-WT + mimic NC group (*p* < 0.01), and the activity of the luciferase reporter in the NTRK2 3′-UTR-WT + miR-429-y mimic group was significantly lower than that in the NTRK2 3′-UTR-WT + mimic NC group (*p* < 0.01) (Figure 7C,D). In contrast, there was no significant difference between the mimic NC and miR-429-y mimic groups in the NTRK2 and LncRNA-MSTRG.19083.1 3′-UTR-MUT group (Figure 7C,D). These data confirm that NTRK2 and LncRNA-MSTRG.19083.1 were specifically targeted and inhibited by miR-429-y in yak LCs.

### 2.8. Transfection Efficiency of miR-429-y and Expression of LncRNA-MSTRG.19083.1 and NTRK2 in Yak Leydig Cells

**Figure 8:**
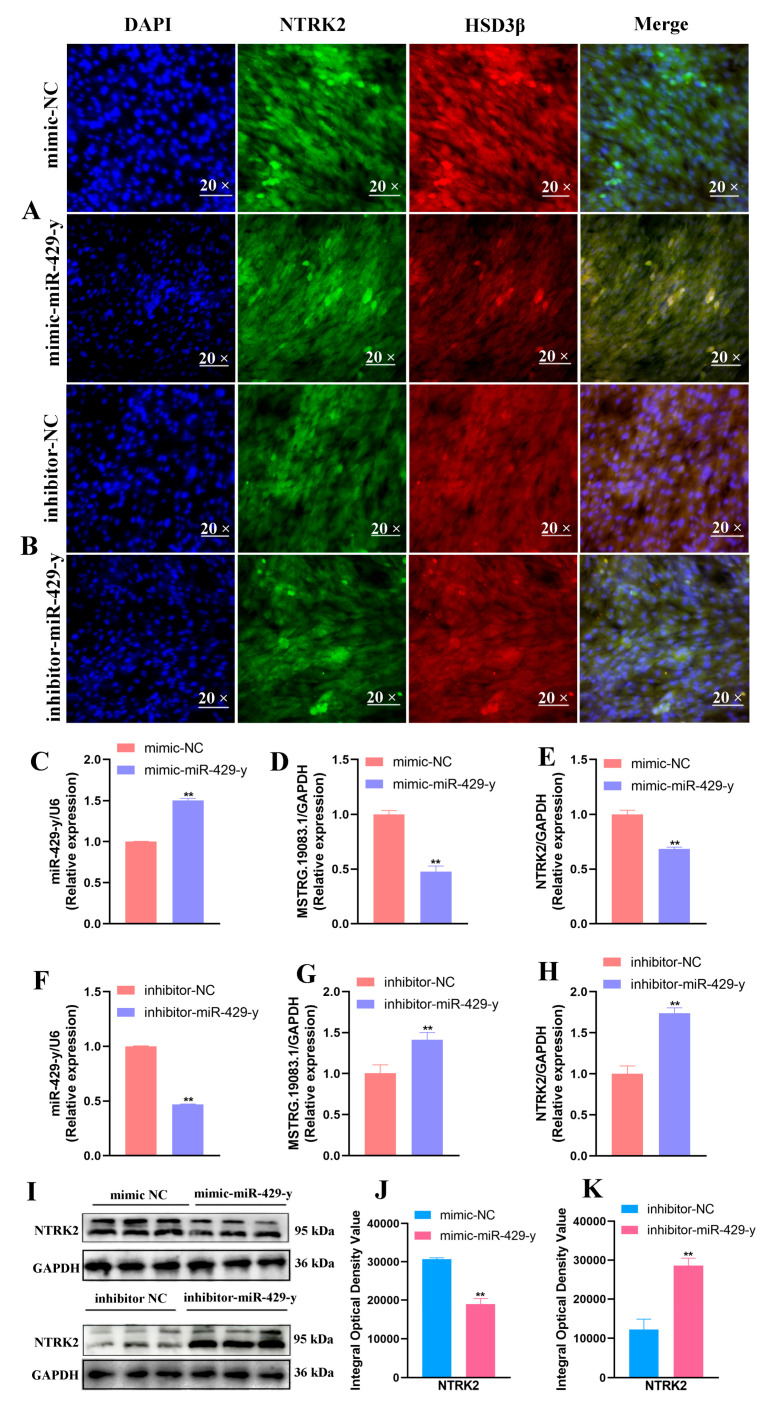
Validation of the targeting relationship between miR-429-y and LncRNA-MSTRG.19083.1 and NTRK2. (**A**,**B**) Localization of NTRK2 protein after transfection with mimic-miR-429-y/inhibitor-miR-429-y in LCs was analyzed by immunofluorescence staining. NTRK2 was colored green, HSD3β is shown in red, and nuclei were counterstained with DAPI (blue); magnification, 20×. (**C**–**F**) mRNA expression of miR-429-y after transfection of 100 nM mimic/inhibitor into LCs for 48 h. Values represent mean ± SD; *n* = 3. ** *p* < 0.01. (**D**,**E**,**G**,**H**) the mRNA expression of LncRNA-MSTRG.19083.1 and NTRK2 after transfection of 100 nM mimic/inhibitor into LCs for 48 h. ** *p* < 0.01. (**I**–**K**) Protein expression of NTRK2 was assessed by Western blotting after transfection of 100 nM mimic/inhibitor into LCs for 48 h (*n* = 3). ** *p* < 0.01.

This study showed that both miR-429-y and inhibitor-miR-429-y had their highest transfection efficiencies at 48 h and 100 nM. Ultimately, 48 h and 100 nM were chosen as optimal conditions for miR-429-y and inhibitor-miR-429-y. The IF analysis showed that when yak LCs were transfected with miR-429-y and inhibitor-miR-429-y, the nuclei were stained blue, NTRK2 was stained green, and HSD3β was stained red (Figure 8A,B and Figure S2). In addition, the qRT-PCR results showed an increased expression of miR-429-y and decreased expression of NTRK2 and LncRNA-MSTRG.19083.1 in the group transfected with miR-429-y compared to the cells transfected with the mimic NC (Figure 8C–E). In contrast, the expression of miR-429-y was decreased, while the expression of NTRK2 and LncRNA-MSTRG.19083.1 was increased following transfection with inhibitor-miR-429-y, compared to the cells transfected with the inhibitor NC (Figure 8F–H). Then, the expression of NTRK2 protein was the same as that of mRNA (Figure 8I–K).

### 2.9. LncRNA-MSTRG.19083.1 Competitive Adsorption miR-429-y Targets NTRK2 to Regulate Circadian Rhythm by cAMP Signaling Pathway in Yak Leydig Cells

**Figure 9:**
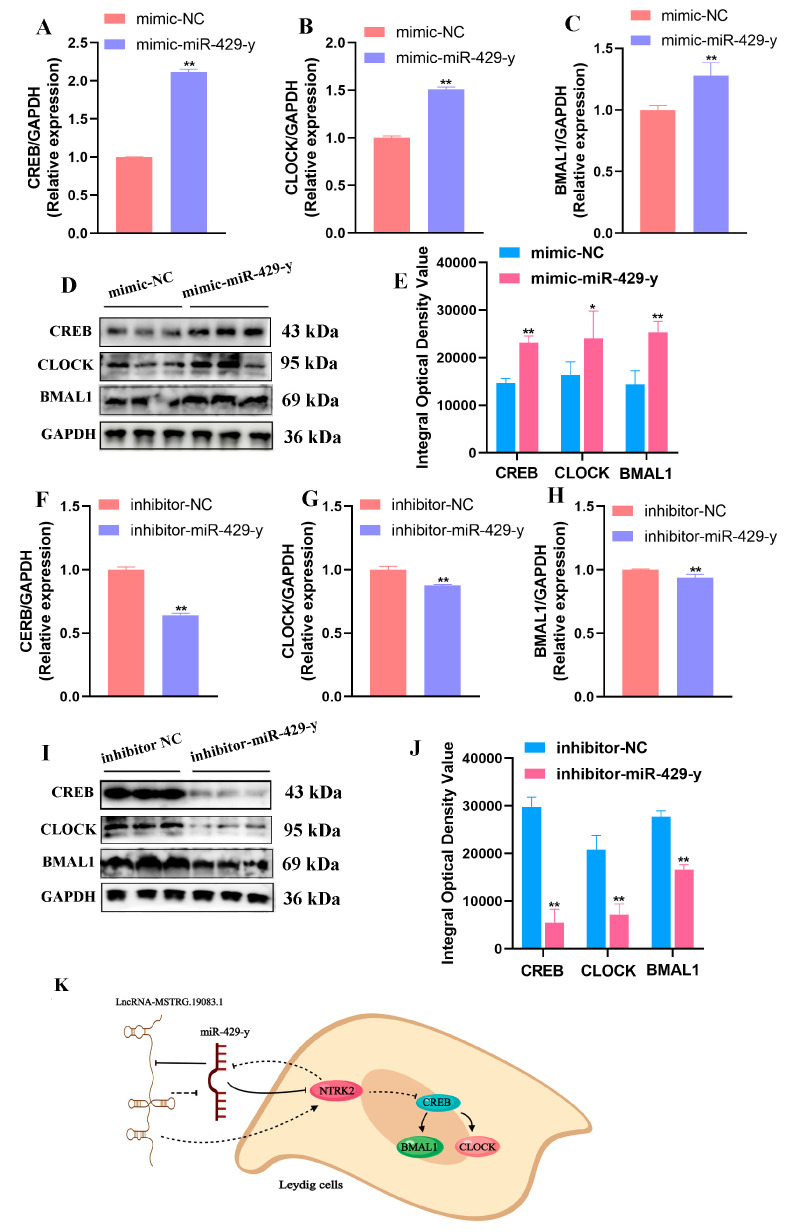
LncRNA-MSTRG.19083.1/miR-429-y targets NTRK2 to mediate the cAMP signaling pathway to regulate circadian rhythm: (**A**–**E**) mRNA and protein expression of CREB, CLOCK, and BAML1 after transfection of 100 nM mimic into LCs for 48 h, * *p* < 0.05, ** *p* < 0.01; (**F**–**J**) mRNA and protein expression of CREB, CLOCK, and BAML1 after transfection of 100 nM inhibitor into LCs for 48 h, ** p < 0.01; (**K**) process model diagram of regulatory mechanism.

We detected the effects of miR-429-y on NTRK2 and further detected the relationship between NTRK2 and circadian rhythm and its regulatory mechanism through qPCR and Western blot. The qPCR results showed that, compared with the mimic NC group, mRNA levels of *CREB*, *CLOCK*, and *BMAL1* in the mimic miR-429-y group were significantly enhanced (*p* < 0.01) (Figure 9A–C). The expression levels of CREB, CLOCK, and BMAL1 proteins in the mimic-miR-429-y group were significantly upregulated (*p* < 0.05) (Figure 9D,E). Specifically, the mRNA levels of *CREB*, *CLOCK*, and *BMAL1* were remarkably decreased in the inhibitor-miR-429-y group compared with those in the inhibitor NC group (*p* < 0.01) (Figure 9F–H). Protein expressions of CREB, CLOCK, and BMAL were significantly downregulated (*p* < 0.05) (Figure 9I,J).

## 3. Materials and Methods

### 3.1. Sample Preparation and Collection

All animals were managed according to the animal care and experimental procedure guidelines approved by the Animal Committee of Gansu Agricultural University (NO: GSAU-Eth-VMC-2023-036). Testis (*n* = 3, Y = 4 years) and cryptorchidism (*n* = 3, Y = 4 years) tissue samples were collected using surgical castration from Wuwei city, Gansu province. One sample (testis and cryptorchid tissue) was rapidly frozen in liquid nitrogen and then stored at −80 °C for the preparation of total RNA and protein, and the other was fixed with 4% paraformaldehyde for about 48 h and then used for making paraffin sections.

### 3.2. Transcriptome Sequencing and Bioinformatics Analysis

The detailed methods for obtaining the strand-specific cDNA library for LncRNA-seq, miRNA-seq, and mRNA-seq in our previous study [33]. The cDNA libraries from the two groups were sequenced on the Illumina HiSeqTM 250 and HiSeqTM 4000 platforms by Gene Denovo Biotechnology Co., Ltd. (Guangzhou, China). According to the manufacturer’s instructions, total RNA was extracted from each testis sample using the Trlquick Reagent (R1100, Solarbio, Beijing, China). The quality and concentration of the RNA were evaluated on a NanoDrop spectrophotometer (Thermo Fisher, Waltham, MA, USA), an Agilent 2100 Bioanalyzer (Agilent Technologies, Santa Clara, CA, USA), and RNase-free agarose gel electrophoresis. All samples had an RNA integrity number (RIN) > 7.5. Fragments per kilobase of exon per million mapped fragments (FPKM) were calculated to determine the miRNA, LncRNA, and mRNA expression levels using StringTie v2.2.0. To identify differentially expressed transcripts across samples or groups, the edgeR package (version 4.4, http://www.bioconductor.org/packages/release/bioc/html/edgeR.html, accessed on 13 April 2024) was used. We identified mRNA, miRNA, and LncRNA with a fold change ≥1 and a false discovery rate (FDR) < 0.05 in a comparison as significant DEGs. To assess functional enrichment, Gene Ontology (GO) Biological Processes term and Kyoto Encyclopedia of Genes and Genomes (KEGG) pathway analyses of mRNAs in the ceRNA network were performed using Cytoscape (version 3.7.1, http://www.geneontology.org/, accessed on 13 April 2024). KEGG is the major public pathway-related database (http://www.kegg.jp/kegg/, accessed on 13 April 2024).

### 3.3. RNA Isolation, cDNA Synthesis, and qRT-PCR

Total RNA was extracted from testis and cryptorchid tissue using the TransZol Up reagent (Invitrogen, Carlsbad, CA, USA), according to the manufacturer’s instructions. After treatment with DNase, RNA was subjected to reverse transcription using a RevertAid First-Strand cDNA Synthesis Kit (TransGen, Beijing, China).

First-strand cDNA for miRNA was synthesized from 500 ng of each total RNA sample using an Evo M-MLV RT Kit with gDNA Clean or miRNA 1st strand cDNA synthesis kit (Accurate Biology, Changsha, China) according to the instructions of the respective kits.

The qPCR was performed using a LightCycler 96 real-time system (Roche, Basel, Switzerland). The qPCR mixture (20 μL total volume) contained 1 μL of cDNA, 2 μL of primer (forward primer 1 μL, reverse primer 1 μL), 10 μL of 2 × TransStart TOP Green qPCR SuperMix (TRANS), and 7 μL of ultrapure Millipore water. The PCR was carried out on a LightCycler 96 system (Roche, Basel, Switzerland). The qPCR amplification was used, and the three stages and amplification conditions were as follows: an initial denaturation at 94 °C for 30 s; denaturation at 94 °C for 30 s followed by 40 cycles; and the optimal annealing temperature for 15 s and 72 °C for 10 s. All PCRs were performed in triplicate. Relative expression values were calculated using the comparative threshold cycle (2^−ΔΔCT^) method. Data are presented as mean value ± SD, *n* = 3. * *p* < 0.05, ** *p* < 0.01. The primers are shown in Table 1. All PCR reactions were performed in triplicate.

### 3.4. H&E Staining

The paraffin sections of yak testes and cryptorchidism were prepared and stained with hematoxylin and eosin (H&E) as previously described [34]. In brief, H&E staining was performed using a Hematoxylin–Eosin/HE Staining Kit (G1120, Solarbio, Beijing, China), which involved dewaxing with xylene, dehydrating with an alcohol gradient, staining with hematoxylin and eosin, dehydrating again, dewaxing with xylene, and, finally, sealing with neutral balsam according to the manufacturer’s instructions. Then, the slides were examined under a light microscope equipped with a digital camera (Olympus, Tokyo, Japan).

### 3.5. Immunohistochemistry Staining

For immunohistochemistry, the paraffin sections were de-paraffinized, rehydrated, and washed thrice with phosphate-buffered saline (PBS). The antigens in the specimens were retrieved with sodium citrate buffer solution. Immunohistochemistry staining was carried out using a HistostainTM-Plus kit (Bioss Biotechnology Co., Ltd., Beijing, China). The sections were incubated with rabbit anti-NTRK2 antibody (1:80; Proteintech, Wuhan, China) overnight at 4 °C in a wet box. Next, positive signals were visualized using a DAB kit (Bioss Biotechnology Co., Ltd., Beijing, China). The representative images were captured using an optical microscope with MvImage software (v3.1, Sunny, Ningbo, China) according to the standard avidin–biotin–peroxidase complex method (ABC Staining System, SABC, BOSTER, Guangzhou, China).

### 3.6. Immunofluorescence Staining

For tissue immunofluorescence, after routine dewaxing and hydration, antigen retrieval in sections was conducted using microwave heating. Sections were treated with an autofluorescence quencher (Servicebio, Wuhan, China), and closed with 5% (*w*/*v*) bovine serum albumin (BSA) for 30 min at room temperature. The sections were incubated with primary antibody (NTRK2, 1:200, Proteintech, Wuhan, China) overnight at 4 °C. After being washed with PBS, sections were incubated with ALEX-conjugated goat anti-rabbit IgG (1:300; Abcam, Burlingame, CA, USA) at room temperature for 1.5 h in the dark. The nuclei were stained with 4′,6-diamidino-2-phenylindole (DAPI). Finally, the anti-fluorescence quencher was used to seal the sections. For immunohistochemistry and immunofluorescence experiments, eight independent biological replicates were carried out, and each biological replicate comprised two technical replicates (LSM800, ZEISS, Oberkochen, Germany).

For cell immunofluorescence, the cells were cultured for 48 h, and a fluorescence microscope (Olympus, Tokyo, Japan) was used to observe the transfection efficiency. The cells were fixed in 4% paraformaldehyde for at least 20 min first and then washed with 4 °C ice-cold PBS three times, treated with 0.1% Triton X-100 for 30 min at room temperature, washed three times, and then incubated with 5% bovine serum albumin (A8010, Solarbio, Beijing, China) for 30 min. Cells were then incubated with rabbit polyclonal antibody for NTRK2 (1:150) at 4 °C overnight. Goat anti-rabbit IgG H&L (Alexa Fluor^®^ 647) secondary antibody (ab169347, 1:200, Abcam, Cambridge, UK) was used at 37 °C for 1 h in the dark. Cell nuclei were stained with 1 μg/mL DAPI. Digital images were acquired using an Olympus DP73 optical microscope (Olympus, Tokyo, Japan).

### 3.7. Cell Culture and Transfection

Leydig cells (LCs) were isolated as previously according to the manufacturer’s instructions from Percoll (BS909-100 mL, Biosharp, Beijing, China), described with minor modifications. Briefly, the testis tissue was minced into a paste and digested with 1% type IV collagenase mixed with 0.25% trypsin for about 0.5 h at 37 °C, after which digestion was terminated with high-glucose DMEM containing 10% FBS. The digested cells were filtered through a 200-mesh screen. Stratified centrifugation was performed according to different density gradients (21%, 36%, 34%, and 60%). After collection and washing, the LCs were cultured in high-glucose DMEM supplemented with 10% fetal bovine serum (FBS) containing streptomycin (50 μg/mL) and penicillin (50 IU/mL) at 37 °C in a 5% CO_2_ atmosphere. The medium was removed and replaced with fresh medium after about 48 h.

Cells were plated in a 35 mm dish prior to transfection to ensure that the monolayer cell density reached an optimal confluency of 80%. The mimic miR-429-y, mimic NC, inhibitor miR-429-y, and inhibitor NC were all purchased from Sangon Biotech (Shanghai, China). Lipofectamine 2000 Reagent (Thermo Fisher Scientific, Carlsbad, CA, USA) was used to transfer mimic or inhibitor according to the manufacturer’s instructions and previous approaches [35]. In brief, for LCs, the optimal ratio of Lipofectamine@2000™ (μL)/mimic or inhibitor (μL) is around 3:1. For each 35 mm dish, 1 μL of mimic or inhibitor was diluted in 50 μL of DMEM, gently pipetted up and down or vortexed briefly to mix. Three microliters of Lipofectamine^@^2000 reagent were diluted in 50 μL of DMEM. Subsequently, diluted Lipofectamine^@^2000 reagent was added to the diluted mimic or inhibitor solution all at once, and immediately pipetted up and down 3–4 times or vortexed briefly to mix and incubated for 15–20 min at room temperature to allow complexes to form. Then, 100 μL of mixture was added dropwise onto the medium in each well, and the mixture was homogenized by gently swirling the plate. It was placed in a cell incubator at 37 °C, replaced with a completely fresh medium after 12–16 h, and the transfection efficiency was determined at 48 h.

### 3.8. Dual-Luciferase Reporter Assays

Wild-type (WT) NTRK2 and LncRNA-MSTRG.19083.1 3′-UTR fragments containing the putative miR-429-y binding site and its corresponding mutant type (Mut) were designed and synthesized by Zebra Biotechnology (Changsha, China) and cloned into the pmirGLO cloning vector between the Mlu I and Xho I cloning sites. In brief, the luciferase reporter plasmids were transfected with the miR-429-y mimic or mimic NC into 293T cells using Lipofectamine 2000. After transfection for 48 h, the luciferase activity was detected using a Dual-Luciferase Reporter Assay Kit (Promega, Madison, WI, USA).

### 3.9. Western Blot

Take an appropriate amount of testicular tissue, add an appropriate amount of liquid nitrogen, and after the tissue has been dehydrated, grind it with a sterile mortar. Then, total protein was isolated from yak testes and SCs using a cell lysis buffer (R0010, Solarbio). A loading buffer was added to the protein samples, and the samples were denatured in a water bath at 100 °C for 10 min. First, 40 μg of the samples were applied to a sodium dodecyl sulfate polyacrylamide gel (SDS-PAGE, 5% + 12%) to confirm the differential protein expression level. Briefly, equal amounts of protein from yak testis and the cryptorchidism group were resolved on SDS-PAGE gels and then transferred electrophoretically onto PVDF membranes. Membranes were then blocked for 2 h at room temperature with 5% non-fat dried milk in PBS containing 0.1% Tween 20. They were then incubated overnight at 4 °C with primary antibodies: GAPDH (6004-1-Ig, 1:5000, Proteintech, China) (Table S1), NTRK2 (13129-1-AP, 1:2500, Proteintech, China), CREB (12208-1-AP, 1:3000, Proteintech, China), BMAL1 (14268-1-AP, 1:3000, Proteintech, China), and CLOCK (ab3517, 1:1000, Abcam, USA). The membranes were then washed three times with PBS containing 0.1% Tween 20 and incubated with goat anti-rabbit IgG conjugated to horseradish peroxidase for 1.5 h at room temperature (1:5000, Bioss, Beijing, China). All immunoblot assays were performed in triplicate at a minimum. Optical densities of bands were quantified, and membranes were scanned using Image-Pro Plus 6.0 (Media Cybernetics Co., Rockville, MD, USA).

### 3.10. Data Analysis

PCR and Western blotting data were expressed as the mean ± SD using Origin 8.6 (OriginLab, Northampton, MA, USA). Data were analyzed using the *t*-test (between two groups) or one-way ANOVA (within multiple groups). A *p*-value of less than 0.05 or 0.01 was statistically significant.

## 4. Discussion

NTRK2, also known as TrkB, is a receptor for brain-derived neurotrophic factor (BDNF) and is a member of the tyrosine kinase receptor superfamily. It is involved not only in the survival, development, and differentiation of neuronal cells and neuroplasticity [36,37] but also in the process of sex hormone secretion, gonad development, and reproductive behavior [38,39]. In mammals, NTRK2 is also associated with sexual drive and satisfaction, and the expression of BDNF/NTRK2 is upregulated during sex, which may help enhance the motivation and execution of sexual activity [40]. Based on transcription combined with GO analysis, NTRK2 may be closely related to male sterility or asthenospermia [41]. We previously revealed that NTRK2 was highly expressed in yak cryptorchids via transcriptomes, and GO analysis showed that NTRK2 was involved in the reproductive process, sexual behavior, and the circadian rhythm process.

In this study, the differential gene NTRK2, which is highly expressed in cryptorchids of yaks, was first screened using transcriptomics of testis and cryptorchids, and the enrichment of GO and KEGG revealed that NTRK2 is mainly involved in reproduction, response stimulation, developmental process, metabolism, behavior, and rhythm, and is also involved in the AKT-PI3K, MAPK, Ras, and neurotrophic factor pathways. At the same time, the qPCR and Western blot revealed the expression profile of NTRK2 in testis and cryptorchid tissues. The IHC results revealed that NTRK2 was mainly expressed in testicular somatic cells (SCs and LCs), which are involved in the reproductive process of spermatogenesis, maturation, and storage. Circadian rhythms are known to be part of an organism’s time-regulating system [42]. Studies have shown that BDNF and its receptor, NTRK2, are particularly important in regulating circadian rhythms; their expression in brain regions, such as the pineal gland, is regulated by photoperiod [43]. For example, BDNF is in an important brain region that regulates circadian rhythms, and the low-affinity neurotrophic factor receptor p75NTR is a clock component [44]. These changes may alter the animal’s activity patterns through NTRK2-mediated signaling pathways, which in turn affect its circadian rhythm.

Through transcriptomic sequencing combined with bioinformatics screening, we found that LncRNA targeting NTRK2 is involved in regulating the circadian rhythm process and reproductive process. Bioinformatics analysis revealed that there were five LncRNA targets and seven miRNA targets in NTRK2, and five LncRNAs competitively bound NTRK2 target genes with seven miRNAs. In the last decade, there have been many studies on the regulation of reproductive physiology by LncRNAs in combination with miRNAs. For example, studies have shown that LINC01963 enhances the chemosensitivity of prostate cancer cells to docetaxel by targeting the miR-216b-5p-TrkB axis [45]. LncPGCAT-1 activates P53 ubiquitination and JNK phosphorylation levels [46]. LncRNA plays a key role in the development of gonads [47]. During the development of ovaries and testes, LncRNAs are involved in regulating the synthesis and secretion of sex hormones, such as the expression of gonadotropin-releasing hormone (GnRH) [48]. In male animals, specific LncRNAs can regulate the process of cell meiosis in the testis [49]. LncRNA-TCL6 promotes early abortion and inhibits placenta implantation via the EGFR pathway [50,51]. In addition, LncRNA can also modify the promoter region of specific genes by recruiting chromatin and affect the expression level of genes related to circadian rhythm. Modifying the transcriptional level of circadian clock genes affects the accurate operation of the circadian clock [52,53,54]. However, the specific regulatory mechanism of LncRNA-targeted NTRK2 involved in the circadian rhythm during the development of cryptorchidism in yaks requires further investigation. In this study, we have transfected mimic-miR-429-y and inhibitor-miR-429-y into yak LCs and then verified the expression of its upstream LncRNA-MSTRG.19083.1 factors and its downstream *NTRK2* genes separately. We found that these constitute a complete endogenous competitive regulatory network that regulates the circadian rhythm of yak LCs via the cAMP signaling pathway (Figure 9K). This is a breakthrough of noncoding RNA in the reproductive regulation of large and rare species, such as yak.

In this study, the ceRNA network of LncRNA-MSTRG.19083.1-miR-429-y-NTRK2 was constructed based on the bioinformatics analysis. From the miRNA expression, the expression levels of upstream LncRNA-MSTRG.19083.1 and downstream NTRK2 were further probed by changing the expression levels of miR-429-y. However, this study did not further validate the effect of NTRK2 on circadian-related markers by knocking down NTRK2 expression levels, and its specific regulatory mechanisms are lacking. Second, this study should be further validated by in vivo animal experiments to reveal the relevance of NTRK2 to cryptorchidism and the molecular mechanism by which NTRK2 modulates circadian rhythms with noncoding RNA.

## 5. Conclusions

Based on RNA-seq, this study revealed the types of LncRNA involved in the development of cryptorchidism in yaks. qPCR, IHC/IF, and Western blot revealed that NTRK2 was involved in regulating the development of cryptorchidism and revealed the target sites of NTRK2. Furthermore, we found that NTRK2 is the target gene of miR-429-y, which is regulated by LncRNA-MSTRG.19083.1. This present study will provide insights for the further exploration of the regulatory mechanism of ncRNAs, especially LncRNAs, which are involved in the circadian rhythm and the occurrence of cryptorchidism in the yaks.

## Figures and Tables

**Table 1:** Information on primer sequences for PCR.

Name	Forward Primer (5′-3′)	Reverse Primer (5′-3′)	Tm (°C)
*NTRK2*	CAGCAACTTACAGCACAT	ATAGACTTTCCCTCCTCC	52
*ADAMTS1*	TTCTTCGTTTTGCAGCCCAA	TCCTCCACAGATGCCACATT	59
*AQP9*	GTTGGGGCTTTGAGGTGTTC	TCTGGTTGTTCTGCCTCCAA	59
*FOXA1*	CAAGCCACCCTACTCGTACA	TCGTTGAAGGAGAGCGAGTG	59
*GPRC5B*	CTCCTGCTCTTCGTGATTGC	CCTTGTCCTTGATGAACGGC	59
*GRIN1*	TCGAGAATATGGCAGGGGTC	TTTTAGGGTCGGGTTCTGCT	59
*HSPB8*	GATACGTGGAGGTGTCTGGT	AACTGCTCTCTCCAAACGGT	59
*IDO1*	ACGTAGGCTTTGCTCTTCCA	CTCGGAGGCCATCAATGTTG	59
*PML*	CCCCAAGAGCCCCATCATAA	TCACTGGACTCACTGCTGTT	59
*SOX10*	CTCAGGACCCTATTACGGCC	TCGTATATACCGGCTGCTCC	59
*TET1*	TTCCAATCAGCCAAACCAGC	ATGTGCTCACTGTCTGACCA	59
*GAPDH*	GCTGGTGCTGAGTATGTGGTG	GCTGACAATCTTGAGGGTGTTG	60
*MSTRG.19083.1*	CCCTGGGCTACAGAACAAA	ACCTGGCTGCGAAACAACT	59
*MSTRG.880.3*	GTCGACCAGCTTCAGACACA	GCCTCAGTCTTCTCCTGTGG	60
*MSTRG.2273.9*	GGCTGAATGTTCAGAGCACA	TTTCCAATCCTGGCATCTTC	57
*MSTRG.13701.2*	GCCGAGGGTTCAGACAGTAG	TTGGACACCCTAGCTCCAAC	60
*MSTRG.2276.3*	TCTTGCTCACACACCCTCAG	AGTGTTCAGGGCAGAGGAGA	60
*MSTRG.19143.1*	CAGGGCATAGGCTTGGATTA	AGGCTCAGTAACAGCCAGGA	59
*MSTRG.18526.1*	ACTAGGGAAGCCCCGAATTA	TGAAAAGCCCCATTCTTTTG	56
*MSTRG.14526.2*	AGCCATTTACCAAGGTGACG	GCTATGCCTTGAGGTTGGAA	58
*MSTRG.7578.2*	GCCCCTTTAGCCCCTTCC	TGGTATTCTGAGTCCACCTTGT	59
*ENSBGRT00000023773*	AAGCTCCAGTTGCTGAAGGA	GCAGTGATGGTCAGCGTAGA	60
*U6*	GGAACGATACAGAGAAGATTAGC	TGGAACGCTTCACGAATTTGCG	60
*miR-429-y*	GCGAATACCTCGGACCCT	Universal reverse *	56
*miR-335-x*	CCAGCGTGTCAAGAGCAATAA	Universal reverse *	51
*miR-7-x*	CCAGTGCGAATACCTCG	Universal reverse *	55
*miR-181-x*	TGTCGGTGAGTGTCGTATCCA	Universal reverse *	60
*miR-6518-z*	AACTGCGAGTCGTATCCA	Universal reverse *	62
*miR-9261-z*	GCGAATACCTCGGACC	Universal reverse *	64
*novel-m0008-3p*	GCCTCTGGGAACACTGTG	Universal reverse *	57
*novel-m0254-3p*	TCTGTCTTCTGTCGTCGTATCCA	Universal reverse *	53

Note: * The universal reverse primer used for RT-qPCR amplification of miRNAs was provided with the kit (Mir-X^TM^ miRNA First-Strand Synthesis Kit).

## Data Availability

The data are available in the Sequence Reads Archive (SRA): https://www.ncbi.nlm.nih.gov/sra/PRJNA1076167 (accessed on 8 January 2024).

## Supplementary Materials

The following supporting information can be downloaded at: https://www.mdpi.com/article/10.3390/ijms252413553/s1.

## References

[1] Goda Y., Mizutani S., Mizutani Y., Kitahara G., Siswandi R., Wakabayashi K., Torisu S., Kaneko Y., Hidaka Y., Osawa T. (2022). Usefulness of computed tomography for cryptorchidism in bulls. J. Vet. Med. Sci..

[2] Zi X.D. (2003). Reproduction in female yaks (*Bos grunniens*) and opportunities for improvement. Theriogenology.

[3] Hutson J.M., Clarke M.C. (2007). Current management of the undescended testicle. Semin. Pediatr. Surg..

[4] Cendron M., Keating M.A., Huff D.S., Koop C.E., Snyder H.M., Duckett J.W. (1989). Cryptorchidism, orchiopexy and infertility: A critical long-term retrospective analysis. J. Urol..

[5] Luo H., Lv W., Tong Q., Jin J., Xu Z., Zuo B. (2021). Functional Non-coding RNA During Embryonic Myogenesis and Postnatal Muscle Development and Disease. Front. Cell Dev. Biol..

[6] Lee J.T. (2012). Epigenetic regulation by long noncoding RNAs. Science.

[7] Luk A.C., Gao H., Xiao S., Liao J., Wang D., Tu J., Rennert O.M., Chan W.Y., Lee T.L. (2015). GermlncRNA: A unique catalogue of long non-coding RNAs and associated regulations in male germ cell development. Database.

[8] Bittman E.L. (2016). Timing in the Testis. J. Biol. Rhythm..

[9] Kadalayil L., Alam M.Z., White C.H., Ghantous A., Walton E., Gruzieva O., Merid S.K., Kumar A., Roy R.P., Solomon O. (2023). Analysis of DNA methylation at birth and in childhood reveals changes associated with season of birth and latitude. Clin. Epigenetics.

[10] Ponjavic J., Ponting C.P., Lunter G. (2007). Functionality or transcriptional noise? Evidence for selection within long noncoding RNAs. Genome Res..

[11] Liang M., Li W., Tian H., Hu T., Wang L., Lin Y., Li Y., Huang H., Sun F. (2014). Sequential expression of long noncoding RNA as mRNA gene expression in specific stages of mouse spermatogenesis. Sci. Rep..

[12] Washietl S., Kellis M., Garber M. (2014). Evolutionary dynamics and tissue specificity of human long noncoding RNAs in six mammals. Genome Res..

[13] Xie M., Utzinger K.S., Blickenstorfer K., Leeners B. (2018). Diurnal and seasonal changes in semen quality of men in subfertile partnerships. Chronobiol. Int..

[14] Ni W., Liu K., Hou G., Pan C., Wu S., Zheng J., Cao J., Chen Q., Huang X. (2019). Diurnal variation in sperm DNA fragmentation: Analysis of 11,382 semen samples from two populations and in vivo animal experiments. Chronobiol. Int..

[15] Ozelci R., Yılmaz S., Dilbaz B., Akpınar F., Akdag Cırık D., Dilbaz S., Ocal A. (2016). Seasonal variation of human sperm cells among 4,422 semen samples: A retrospective study in Turkey. Syst. Biol. Reprod. Med..

[16] Bass J., Takahashi J.S. (2010). Circadian integration of metabolism and energetics. Science.

[17] Mohawk J.A., Green C.B., Takahashi J.S. (2012). Central and peripheral circadian clocks in mammals. Annu. Rev. Neurosci..

[18] Potter G.D., Cade J.E., Grant P.J., Hardie L.J. (2016). Nutrition and the circadian system. Br. J. Nutr..

[19] Ding J., Chen P., Qi C. (2024). Circadian rhythm regulation in the immune system. Immunology.

[20] Kume K., Zylka M.J., Sriram S., Shearman L.P., Weaver D.R., Jin X., Maywood E.S., Hastings M.H., Reppert S.M. (1999). mCRY1 and mCRY2 are essential components of the negative limb of the circadian clock feedback loop. Cell.

[21] Cheng S., Liang X., Wang Y., Jiang Z., Liu Y., Hou W., Li S., Zhang J., Wang Z. (2016). The circadian Clock gene regulates acrosin activity of sperm through serine protease inhibitor A3K. Exp. Biol. Med..

[22] Peruquetti R.L., de Mateo S., Sassone-Corsi P. (2012). Circadian proteins CLOCK and BMAL1 in the chromatoid body, a RNA processing granule of male germ cells. PLoS ONE.

[23] Qin X., Zhao Y., Zhang T., Yin C., Qiao J., Guo W., Lu B. (2022). TrkB agonist antibody ameliorates fertility deficits in aged and cyclophosphamide-induced premature ovarian failure model mice. Nat. Commun..

[24] Levine E.S., Dreyfus C.F., Black I.B., Plummer M.R. (1995). Brain-derived neurotrophic factor rapidly enhances synaptic transmission in hippocampal neurons via postsynaptic tyrosine kinase receptors. Proc. Natl. Acad. Sci. USA.

[25] Asadian N., Parsaie H., Vafaei A.A., Dadkhah M., Omoumi S., Sedaghat K. (2022). Chronic light deprivation induces different effects on spatial and fear memory and hippocampal BDNF/TRKB expression during light and dark phases of rat diurnal rhythm. Behav. Brain Res..

[26] Batra N.V., DeMarco R.T., Bayne C.E. (2021). A narrative review of the history and evidence-base for the timing of orchidopexy for cryptorchidism. J. Pediatr. Urol..

[27] Mitsui T. (2021). Effects of the prenatal environment on cryptorchidism: A narrative review. Int. J. Urol..

[28] Camerino C., Conte E., Cannone M., Caloiero R., Fonzino A., Tricarico D. (2016). Nerve Growth Factor, Brain-Derived Neurotrophic Factor and Osteocalcin Gene Relationship in Energy Regulation, Bone Homeostasis and Reproductive Organs Analyzed by mRNA Quantitative Evaluation and Linear Correlation Analysis. Front. Physiol..

[29] Khan F.A., Gartley C.J., Khanam A. (2018). Canine cryptorchidism: An update. Reprod. Domest. Anim. Zuchthyg..

[30] Schulster M., Bernie A.M., Ramasamy R. (2016). The role of estradiol in male reproductive function. Asian J. Androl..

[31] Jia Y., Liu Y., Wang P., Liu Z., Zhang R., Chu M., Zhao A. (2024). NTRK2 Promotes Sheep Granulosa Cells Proliferation and Reproductive Hormone Secretion and Activates the PI3K/AKT Pathway. Animals.

[32] Hawley W.R., Mosura D.E. (2019). Sexual motivation in male rats is modulated by tropomyosin receptor kinase B (TrkB). Behav. Neurosci..

[33] Yan Q., Wang Q., Zhang Y., Yuan L., Hu J., Zhao X. (2024). The Novel-m0230-3p miRNA Modulates the CSF1/CSF1R/Ras Pathway to Regulate the Cell Tight Junctions and Blood-Testis Barrier in Yak. Cells.

[34] Li T., Lu Z., Luo R., Gao J., Zhao X., Ma Y. (2018). Expression and cellular localization of double sex and mab-3 related transcription factor 1 in testes of postnatal Small-Tail Han sheep at different developmental stages. Gene.

[35] Salama S.A., Arab H.H., Hassan M.H., Al Robaian M.M., Maghrabi I.A. (2019). Cadmium-induced hepatocellular injury: Modulatory effects of γ-glutamyl cysteine on the biomarkers of inflammation, DNA damage, and apoptotic cell death. J. Trace Elem. Med. Biol..

[36] Rask-Andersen M., Almén M.S., Olausen H.R., Olszewski P.K., Eriksson J., Chavan R.A., Levine A.S., Fredriksson R., Schiöth H.B. (2011). Functional coupling analysis suggests link between the obesity gene FTO and the BDNF-NTRK2 signaling pathway. BMC Neurosci..

[37] Wu H., Uchimura K., Donnelly E.L., Kirita Y., Morris S.A., Humphreys B.D. (2018). Comparative Analysis and Refinement of Human PSC-Derived Kidney Organoid Differentiation with Single-Cell Transcriptomics. Cell Stem Cell.

[38] Gao S., Li C., Xu Y., Chen S., Zhao Y., Chen L., Jiang Y., Liu Z., Fan R., Sun L. (2017). Differential expression of microRNAs in TM3 Leydig cells of mice treated with brain-derived neurotrophic factor. Cell Biochem. Funct..

[39] Liu B., Liu Y., Li S., Chen P., Zhang J., Feng L. (2023). BDNF promotes mouse follicular development and reverses ovarian aging by promoting cell proliferation. J. Ovarian Res..

[40] Sanna F., Poddighe L., Serra M.P., Boi M., Bratzu J., Sanna F., Corda M.G., Giorgi O., Melis M.R., Argiolas A. (2019). c-Fos, ΔFosB, BDNF, trkB and Arc Expression in the Limbic System of Male Roman High- and Low-Avoidance Rats that Show Differences in Sexual Behavior: Effect of Sexual Activity. Neuroscience.

[41] Li L., Chen S. (2021). Screening, identification and interaction analysis of key MicroRNAs and genes in Asthenozoospermia. Int. J. Med. Sci..

[42] Ishii T., Warabi E., Mann G.E. (2019). Circadian control of BDNF-mediated Nrf2 activation in astrocytes protects dopaminergic neurons from ferroptosis. Free Radic. Biol. Med..

[43] Sompol P., Liu X., Baba K., Paul K.N., Tosini G., Iuvone P.M., Ye K. (2011). N-acetylserotonin promotes hippocampal neuroprogenitor cell proliferation in sleep-deprived mice. Proc. Natl. Acad. Sci. USA.

[44] Ishii T., Warabi E., Mann G.E. (2018). Circadian control of p75 neurotrophin receptor leads to alternate activation of Nrf2 and c-Rel to reset energy metabolism in astrocytes via brain-derived neurotrophic factor. Free Radic. Biol. Med..

[45] Xing Z., Li S., Xing J., Yu G., Wang G., Liu Z. (2022). Silencing of LINC01963 enhances the chemosensitivity of prostate cancer cells to docetaxel by targeting the miR-216b-5p/TrkB axis. Lab. Investig..

[46] Zuo Q., Jin J., Jin K., Zhou J., Sun C., Song J., Chen G., Zhang Y., Li B. (2020). P53 and H3K4me2 activate N6-methylated LncPGCAT-1 to regulate primordial germ cell formation via MAPK signaling. J. Cell Physiol..

[47] Li Y., Zhai H., Tong L., Wang C., Xie Z., Zheng K. (2023). LncRNA Functional Screening in Organismal Development. Noncoding RNA.

[48] Warita K., Mitsuhashi T., Ohta K., Suzuki S., Hoshi N., Miki T., Takeuchi Y. (2013). In vitro evaluation of gene expression changes for gonadotropin-releasing hormone 1, brain-derived neurotrophic factor and neurotrophic tyrosine kinase, receptor, type 2, in response to bisphenol A treatment. Congenit. Anom..

[49] Wang Y., Zhang L., Kong R., Hu C., Zhao Z., Wu Y., Zuo Q., Li B., Zhang Y.N. (2023). Jun-mediated lncRNA-IMS promotes the meiosis of chicken spermatogonial stem cells via gga-miR-31-5p/stra8. Mol. Reprod. Dev..

[50] Liu L.P., Gong Y.B. (2018). LncRNA-TCL6 promotes early abortion and inhibits placenta implantation via the EGFR pathway. Eur. Rev. Med. Pharmacol. Sci..

[51] Aljubran F., Nothnick W.B. (2021). Long non-coding RNAs in endometrial physiology and pathophysiology. Mol. Cell Endocrinol..

[52] Fan Z., Zhao M., Joshi P.D., Li P., Zhang Y., Guo W., Xu Y., Wang H., Zhao Z., Yan J. (2017). A class of circadian long non-coding RNAs mark enhancers modulating long-range circadian gene regulation. Nucleic Acids Res..

[53] Sánchez-Retuerta C., Suaréz-López P., Henriques R. (2018). Under a New Light: Regulation of Light-Dependent Pathways by Non-coding RNAs. Front. Plant Sci..

[54] Wang Q., Zhang Q., Gan Z., Li H., Yang Y., Zhang Y., Zhao X. (2020). Screening for reproductive biomarkers in Bactrian camel via iTRAQ analysis of proteomes. Reprod. Domest. Anim. Zuchthyg..

