# Supplementary material for: LncRNA-MSTRG.19083.1 Targets NTRK2 as a miR-429-y Sponge to Regulate Circadian Rhythm via the cAMP Pathway in Yak Testis and Cryptorchidism
Source: Int J Mol Sci. 2024 Dec 18;25(24):13553. doi: 10.3390/ijms252413553 (PMC11678581; doi:10.3390/ijms252413553)
Supplement: Supplementary file 1 [file ijms-25-13553-s001.zip › Figures S1 and S2.pdf]

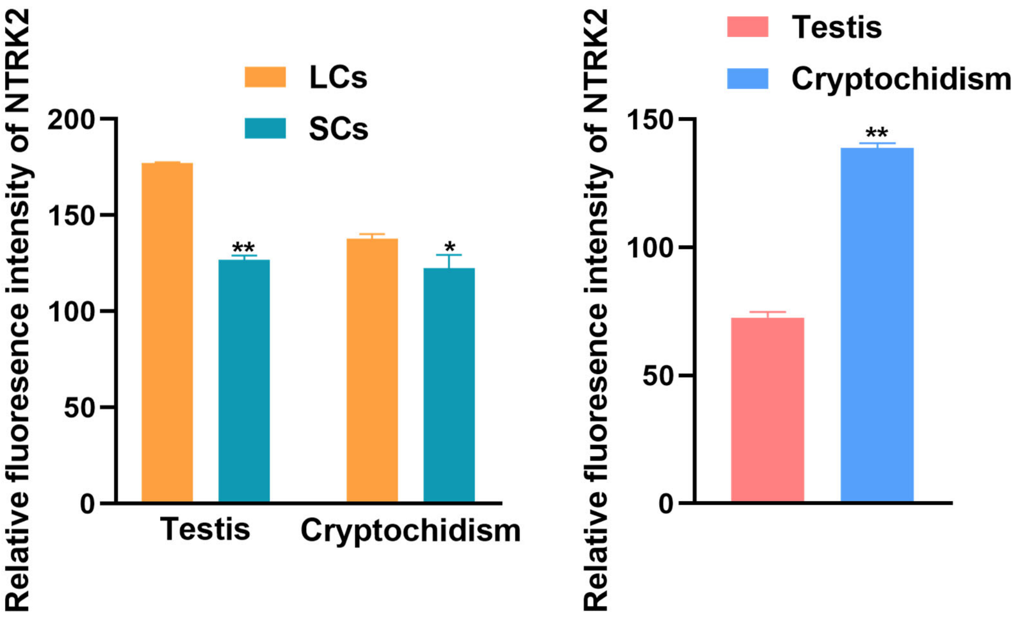

(**A**) (**B**)

Figure S1. Immunofluorescence analysis: (**A**) Immunofluorescence analysis of NTRK2 in yak testis and cryptorchid LCs and SCs; (**B**) Immunofluorescence analysis of NTRK2 in LCs of yak testis and cryptorchid testis. * p < 0.05. ** p < 0.01.

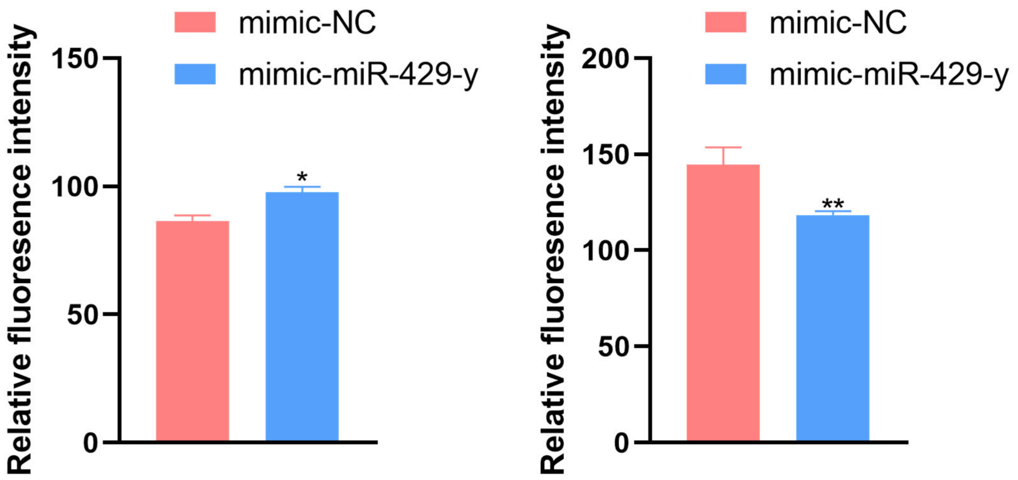

(**A**) (**B**)

Figure S2. Immunofluorescence analysis: (**A**) I Immunofluorescence analysis of mimic-NC and mimic-miR-429-y; (**B**) Immunofluorescence analysis of inhibitor-NC and inhibitor-miR-429-y. * p < 0.05. ** p < 0.01.
