# Supplementary material for: LncRNA-MSTRG.19083.1 Targets NTRK2 as a miR-429-y Sponge to Regulate Circadian Rhythm via the cAMP Pathway in Yak Testis and Cryptorchidism
Source: Int J Mol Sci. 2024 Dec 18;25(24):13553. doi: 10.3390/ijms252413553 (PMC11678581; doi:10.3390/ijms252413553)
Supplement: Supplementary file 1 [file ijms-25-13553-s001.zip › Table S1.pdf]

Table S1. Information on antibodies.

Name	Manufacturer	Cat No.	Type of experiment	Dilution ratio	Diluent solvent	Time	Temperature
NTRK2	Proteintech	13129-1-AP	WB	1:2500	PBST	13h	4℃
			IHC	1:80	PBS	12h	4℃
			IF	1:200	PBS	12h	4℃
CREB	Proteintech	12208-1-AP	WB	1:3000	PBST	13h	4℃
BMAL1	Proteintech	14268-1-AP	WB	1:3000	PBST	13h	4℃
CLOCK	Abcam	ab3517	WB	1:1000	PBST	13h	4℃
GAPDH	Proteintech	6004-1-Ig	WB	1:5000	PBST	13h	4℃
HRP-conjugated Goat Anti-Rabbit IgG(H+L)	Proteintech	SA00001-2	WB	1:5000	PBST	1.5h	37℃
Goat Anti-Rabbit IgG H&L (Alexa Fluor® 647)	Abcam	ab150079	IF	1:300	PBS	1.5h	Room temperature
Goat Anti-Rabbit IgG H&L (Alexa Fluor® 488)	Abcam	ab150077	IF	1:300	PBS	1.5h	Room temperature
HSD3β	Abmart	PS05629S	IF	1:100	PBS	12h	4℃
β-tubulin	Abmart	M20005S	IF	1:300	PBS	12h	4℃
